# Effects of *O*‐methylated (−)‐epigallocatechin gallate (EGCG) on LPS‐induced osteoclastogenesis, bone resorption, and alveolar bone loss in mice

**DOI:** 10.1002/2211-5463.12340

**Published:** 2017-11-15

**Authors:** Tsukasa Tominari, Ryota Ichimaru, Shosei Yoshinouchi, Chiho Matsumoto, Kenta Watanabe, Michiko Hirata, Florian M.W. Grundler, Masaki Inada, Chisato Miyaura

**Affiliations:** ^1^ Department of Biotechnology and Life Science Tokyo University of Agriculture and Technology Koganei Japan; ^2^ Institute of Global Innovation Research Tokyo University of Agriculture and Technology Koganei Japan; ^3^ Institute of Crop Science and Resource Conservation University of Bonn Germany

**Keywords:** bone resorption, lipopolysaccharide, *O*‐methylated EGCG, periodontitis

## Abstract

(−)‐Epigallocatechin‐3‐*O*‐gallate (EGCG), present in green tea, exhibits antioxidant and antiallergy effects. EGCG3″Me, a 3‐*O*‐methylated derivative of EGCG, has been reported to show similar biological functions; the inhibitory activity of EGCG3″Me in a mouse allergy model was more potent than that of EGCG, probably due to the efficiency of absorption from the intestine. However, the functional potency of these EGCGs is controversial in each disease model. We previously observed that EGCG suppressed inflammatory bone resorption and prevented alveolar bone loss in a mouse model of periodontosis. In this study, we examined the role of EGCG3″Me in bone resorption using a mouse model of periodontitis. Lipopolysaccharide (LPS)‐induced osteoclast formation was suppressed by adding EGCG3″Me to cocultures of osteoblasts and bone marrow cells, and LPS‐induced bone resorption was also inhibited by EGCG3″Me in calvarial organ cultures. EGCG3″Me acted on osteoblasts and suppressed prostaglandin E (PGE) production, which is critical for inflammatory bone resorption, by inhibiting the expression of COX‐2 and mPGES‐1, key enzymes for PGE synthesis. In osteoclast precursor macrophages, EGCG3″Me suppressed RANKL‐dependent differentiation into mature osteoclasts. In a mouse model of periodontitis, LPS‐induced bone resorption was suppressed by EGCG3″Me in organ culture of mouse alveolar bone, and the alveolar bone loss was further attenuated by the treatment of EGCG3″Me in the lower gingiva *in vivo*. EGCG3″Me may be a potential natural compound for the protection of inflammatory bone loss in periodontitis.

**Abbreviations**
BMMs: bone marrow macrophages
COX‐2: cyclooxygenase‐2
EGCG: (−)‐epigallocatechin‐3‐*O*‐gallate
EGCG3″Me: *O*‐methylated (−)‐epigallocatechin gallate
IL‐1: interleukin‐1
LPS: lipopolysaccharide
mPGES‐1: membrane‐bound prostaglandin E synthase‐1
PGE: prostaglandin E
RANKL: receptor activator of NF‐κB ligand
sRANKL: soluble RANK ligand
TLR4: toll‐like receptor 4
TNF‐α: tumor necrosis factor‐α
TRAP: tartrate‐resistant acid phosphatase

Bone remodeling is precisely regulated by a balance of osteoclastic bone resorption and osteoblastic bone formation. Receptor activator of NF‐κB ligand (RANKL) is expressed on the cell surface of osteoblasts in response to bone‐resorbing factors, such as lipopolysaccharide (LPS), interleukin (IL)‐1, and tumor necrosis factor (TNF)‐α 1, 2, 3. RANK is expressed on osteoclast precursor cells, and osteoblast‐derived RANKL promotes the differentiation of precursor cells into mature osteoclasts 4, 5. Inflammatory bone‐resorbing factors, such as LPS and IL‐1, stimulate the mRNA expression of cyclooxygenase (COX)‐2 and membrane‐bound prostaglandin (PG) E synthase (mPGES)‐1, leading to PGE_2_ production by osteoblasts 6, 7, 8. We previously reported that PGE_2_ production is essential for inflammatory bone resorption, and PGE_2_ is recognized by its receptor EP4 in osteoblasts, inducing the expression of RANKL and resulting in osteoclast differentiation 8, 9.

Periodontitis is an inflammatory bone disease caused by the infection of mixed Gram‐negative bacteria. The progression of periodontitis results in alveolar bone destruction and tooth loss. LPS is an outer membrane component of Gram‐negative bacteria and contributes to the pathogenesis of periodontitis via toll‐like receptor (TLR) 4 signaling. We established a novel mouse model for periodontitis and reported that the LPS‐induced alveolar bone loss was attenuated in mPGES‐1‐deficient mice, suggesting that mPGES‐1‐mediated PGE_2_ synthesis is essential for LPS‐mediated bone loss in periodontitis 7.

(−)‐Epigallocatechin‐3‐*O‐*gallate (EGCG) is a green tea‐derived catechin that exerts a variety of beneficial effects, including antioxidative and antibacterial activities 10, 11. A methylated derivative of EGCG, 3‐*O*‐methylated EGCG (EGCG3″Me), is also reported to exert biological activity, such as antiallergic effects 12. Suzuki *et al*. 13 reported that the inhibitory activity of EGCG3″Me was higher than that of EGCG in a mouse type IV allergy model, probably due to the absorption efficiency from the intestine. However, Yano *et al*. 14 examined the suppressive effects of EGCG3″Me and EGCG in the expression of IgE receptor in human basophilic cells and conversely found that the effects of EGCG3″Me were lower than those of EGCG. Therefore, the biological roles of EGCG and EGCG3″Me are controversial in different disease models, and different rates of intestinal absorption may be involved in these compounds’ respective biological potencies.

We previously reported that EGCG suppresses LPS‐induced bone resorption via the negative regulation of PGE_2_ production by osteoblasts and that EGCG restored alveolar bone loss induced by LPS in a mouse model of periodontitis 15. However, the effects of EGCG3″Me on bone resorption are not known. In this study, we examined the effects of EGCG3″Me on RANKL‐dependent osteoclast differentiation and LPS‐induced inflammatory bone resorption. We also elucidated the effects of EGCG3″Me on alveolar bone destruction in a mouse model of periodontitis.

## Materials and methods

### Animals and reagents

Newborn, 5‐week‐old, and 6‐week‐old mice of the *ddY* strain were obtained from Japan SLC, Inc. (Shizuoka, Japan). All procedures were performed in accordance with the institutional guidelines for animal research. Highly purified EGCG3″Me (purity: ≥98%) was obtained from Tokiwa Phytochemical Co., Ltd. (Chiba, Japan). LPS from *Escherichia coli* was provided by Sigma‐Aldrich Co. LLC. (St. Louis, MO, USA). Recombinant human soluble RANK ligand (sRANKL) was purchased from Peprotech Co., Ltd. (Rocky Hill, NJ, USA).

### Culture of primary mouse osteoblastic cells

Primary osteoblastic cells (POBs) were isolated from newborn mouse calvariae after five routine sequential digestions with 0.1% collagenase (Roche Diagnostics GmbH, Mannheim, Germany) and 0.2% dispase (Roche Applied Science, Mannheim, Germany) as described previously 7. POBs were collected from fractions 2–4 and combined, and cultured for 3 days in α‐modified MEM (αMEM) supplemented with 10% fetal bovine serum (FBS) at 37 °C under 5% CO_2_ in air. After POBs reached to confluence, they were trypsinized, counted, and used for the respective experiment.

### Cocultures of mouse bone marrow cells and osteoblasts

Bone marrow cells (BMCs) were isolated from tibiae of 6‐week‐old mice as reported previously 4, 6. BMCs (2 × 10^6^ cells) and POBs (1 × 10^4^ cells) were cocultured with or without LPS (1 ng·mL^−1^) and EGCG3″Me (3–30 μm) in αMEM containing 10% FBS for 7 days. The cells adhering to the well surface were stained for tartrate‐resistant acid phosphatase (TRAP), and TRAP‐positive multinucleated cells containing ≥3 nuclei per cell were counted as osteoclasts.

### Bone‐resorbing activity in organ cultures of mouse calvariae

Newborn mouse calvariae were collected and precultured for 24 h in BGJb medium with 0.1% bovine serum albumin (BSA) at 37 °C under 5% CO_2_ in the air. Calvariae were treated with LPS (1 μg·mL^−1^) in the presence or absence of EGCG3″Me and cultured for 5 days. The bone‐resorbing activity was elucidated by measuring the increased medium calcium, as reported previously 8.

### Measurement of the PGE_2_ content

Primary osteoblastic cells were cultured in αMEM containing 10% FBS, and the concentration of PGE_2_ in the conditioned medium was measured using an enzyme immunoassay system (EIA) (GE Healthcare UK, Ltd., Little Chalfont, UK). The cross‐reactivity of the antibody in the EIA was calculated as follows: PGE_2_, 100%; PGE_1_, 7.0%; 6‐keto‐PGF_1α_, 5.4%; PGF_2α_, 4.3%; and PGD_2_, 1.0%.

### Real‐time PCR analysis

Primary osteoblastic cells were cultured for 24 h in αMEM with 1% FBS with or without LPS (1 ng·mL^−1^) and EGCG3″Me (30 μm). Total RNA was isolated using ISOGEN (Nippon Gene Co., Ltd., Toyama, Japan), and cDNA was prepared from RNA via reverse transcription. For a real‐time PCR analysis, 5 μg of RNA was mixed with SsoAdvanced SYBR green supermix (Bio‐Rad, Hercules, CA, USA) and a PCR primer pair, and a quantitative PCR (qPCR) analysis was performed using ΔΔCq (Cq; quantification cycle) methods 16. The expression level of the target gene was normalized to that of the reference gene, β‐actin, and the ratio of the expression of the test group was compared with that of the control group.

The primer sequences for qPCR, as used in the previous studies 6, 7, 15, are shown in Table 1. The results are shown as the relative fold expression compared with the control.

**Table 1:** Primers used in this work

Genes	Forward	Reverse
mouse Rankl (NM_011613.3)	5′‐aggctgggccaagatctcta‐3′	5′‐gtctgtaggtacgcttcccg‐3′
mouse Cox‐2 (NM_011198.4)	5′‐gggagtctggaacattgtgaa‐3′	5′‐gtgcacatt gtaagtaggtggact‐3′
mouse mPges‐1 (NM_022415.3)	5′‐gcacactgctggtcatcaag‐3′	5′‐acgtttcagcgcatcctc‐3′
mouse Nfatc1 (NM_001164109.1)	5′‐agtctctttccccgacatca‐3′	5′‐cacctcgatccgaagctc‐3′
mouse Ctsk (NM_007802.4)	5′‐gcctagcgaacagattctcaa‐3′	5′‐cactgggtgtccagcattt‐3′
mouse Oscar (NM_001290377.1)	5′‐cttccccagcccttactacc‐3′	5′‐gagttgccacacagcatcac‐3′
mouse β‐actin (NM_007393.5)	5′‐ccccattgaacatggcattg‐3′	5′‐acgaccagaggcatacagg‐3′

### IκB kinase assay

The IκB kinase (IKK) activity of IKKβ was elucidated *in vitro* using test tubes with or without EGCG3″Me (1 mm) using the Cyclex IKKα and β Assay/Inhibitor Screening Kit (CycLex Co., Ltd., Nagano, Japan) with the IKKβ, IκBα, and anti‐phospho‐IκBα antibodies.

### Dual‐luciferase reporter assay

Plasmid pNFκB‐TA‐Luc (0.4 μg) contained four tandem copies of the NF‐κB consensus sequence with the firefly luciferase reporter gene (Clontech Laboratories, Inc., Mountain View, CA, USA) and the pGL4.74[hLuc/TK] plasmid (40 ng) contained the renilla luciferase reporter gene (Promega Corp., Fitchburg, WI, USA) as an internal control reporter vector. Both plasmids were transfected into POBs in cultures using Lipofectamine 2000 (Thermo Fisher Scientific Inc., Waltham, MA, USA) and cultured for 24 h with or without LPS (1 ng·mL^−1^) and EGCG3″Me (30 μm). The luciferase activity was measured using the Dual‐luciferase Reporter Assay System (Promega Corp.) with an ARVO MX multilabel/luminescence counter (Perkin Elmer Corp., Waltham, MA, USA).

### Osteoclast differentiation from macrophages

Bone marrow macrophages (BMMs) were prepared by three days’ culture with M‐CSF (100 ng·mL^−1^), and cultured for 5 days with or without sRANKL (100 ng·mL^−1^). RAW264.7 cells (a murine macrophage cell line) were also cultured for 5 days with or without sRANKL (100 ng·mL^−1^). The TRAP‐positive multinucleated cells that contained ≥3 nuclei per cell were counted as osteoclasts.

### Organ cultures of mouse alveolar bone

Mandibular alveolar bones were collected from 5‐week‐old mice under a microscope and cultured for 24 h in BGJb medium with 0.1% BSA at 37 °C under 5% CO_2_ in the air. After 24 h, alveolar bones were treated with LPS (5 μg·mL^−1^) in the presence or absence of EGCG3″Me and cultured for 5 days. The bone‐resorbing activity was determined by measuring the concentration of calcium in the conditioned medium.

### Measurement of alveolar bone mineral density in mice

Lipopolysaccharide (25 μg per mouse) with or without EGCG3″Me (100 μg per mouse) was injected into the lower gingiva of mice on days 0, 2, and 4 to establish an experimental model of periodontitis. The mandibular alveolar bones were collected on day 7, and the bone mineral density (BMD) of the alveolar bone was measured by dual X‐ray absorptiometry (model DCS‐600R; Aloka, Tokyo, Japan), as reported previously 7.

### Statistical analyses

Data were analyzed using one‐way ANOVA, followed by Tukey's test for *post hoc* analysis. All data are presented as the means ± SEM, and all statistical analyses were performed using ibm spss Statistics Ver.23 software (Armonk, NY, USA).

## Results

### Effects of EGCG3″Me on LPS‐induced osteoclast formation and bone resorption

**Figure 1:**
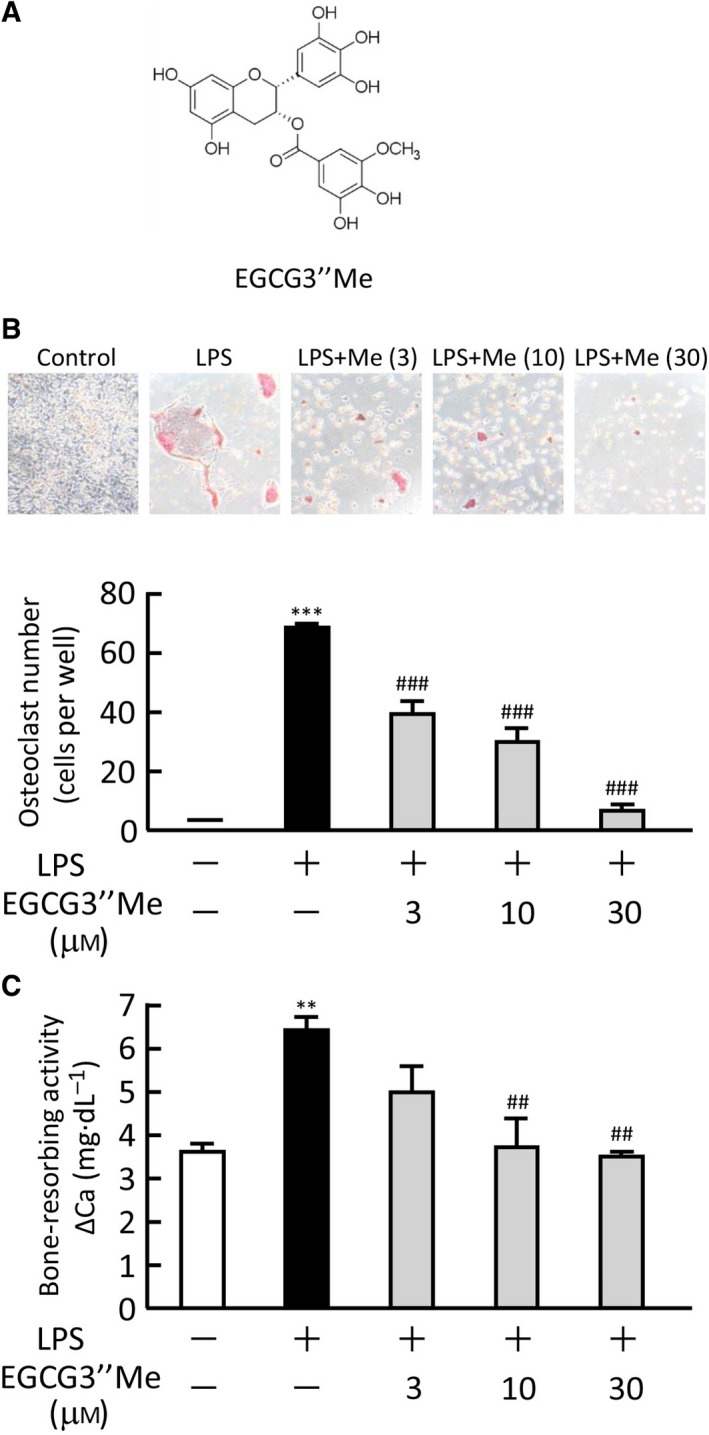
The effects of EGCG3″Me on the LPS‐induced osteoclast differentiation and bone resorption. (A) The chemical structure of EGCG3″Me. (B) Mouse POBs and BMCs were cocultured for 7 days with LPS (1 ng·mL^−1^) and EGCG3″Me (3–30 μm). TRAP‐positive multinuclear osteoclasts were defined as osteoclasts. The upper panel shows the TRAP‐stained cells in each culture group. The data are expressed as the means ± SEM of three wells. (C) Mouse calvariae were cultured for 5 days with LPS (1 μg·mL^−1^) and EGCG3″Me (3–30 μm). The bone‐resorbing activity was measured based on the calcium in the medium. The data are expressed as the means ± SEM of four cultures. A significant difference between the two groups was indicated; **P < 0.01 and ***P < 0.001 vs. control, ^##^ *P *< 0.01 and ^###^ *P *< 0.001 vs. LPS.

The structure of EGCG3″Me is shown in Fig. 1A. To examine the effects of EGCG3″Me on osteoclast differentiation, POBs and BMCs were cocultured in the presence or absence of LPS (1 ng·mL^−1^) and various doses of EGCG3″Me. LPS induced the formation of TRAP‐positive osteoclasts in the coculture, but adding EGCG3″Me (3–30 μm) dose dependently suppressed the LPS‐induced osteoclast formation (Fig. 1B). In organ culture of mouse calvariae, EGCG3″Me (3–30 μm) clearly inhibited the bone‐resorbing activity induced by LPS (Fig. 1C).

### PGE2 production and the mRNA expression of COX‐2, mPGES‐1, and RANKL in osteoblasts

**Figure 2:**
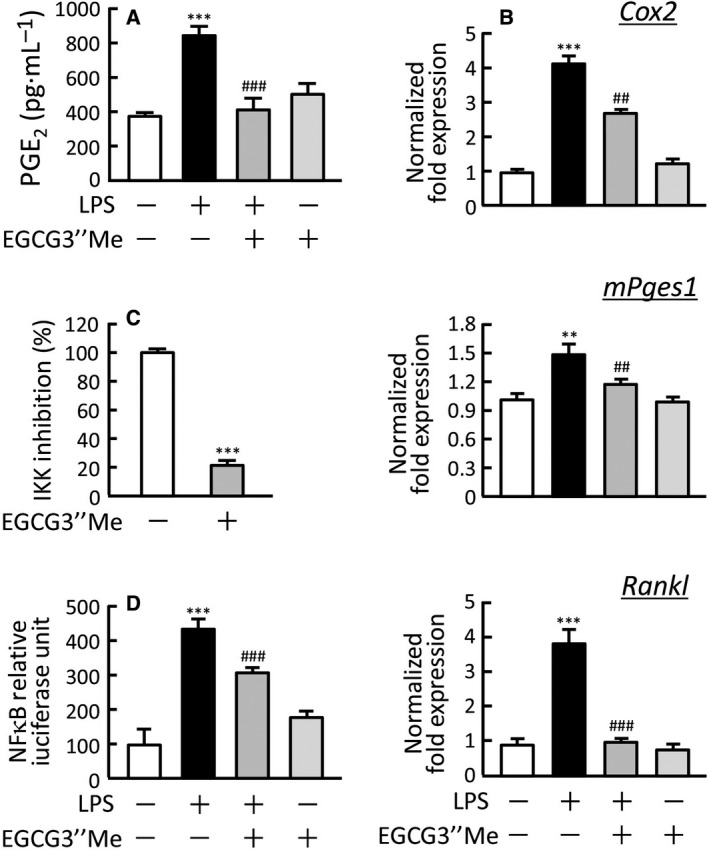
The effects of EGCG3″Me on PGE_2_ production and the mRNA expression of related genes in osteoblasts. (A) The levels of PGE_2_ in cultured medium of osteoblasts. POBs were cultured for 24 h with or without LPS (1 ng·mL^−1^) and EGCG3″Me (30 μm), and the conditioned medium was collected to measure the levels of PGE_2_. (B) The mRNA expression of COX‐2 (*Cox2*), mPGES‐1 (*mPges1*), and RANKL (*Rankl*) in osteoblasts was analyzed using real‐time PCR. POBs were cultured for 24 h with or without LPS (1 ng·mL^−1^) and EGCG3″Me (30 μm), and total RNA was isolated for real‐time PCR. (C) IKK activity was determined *in vitro* with or without EGCG3″Me (1 mm) by the IKK activity assay kit using IKKβ, IκBα, and anti‐phospho‐IκBα antibody. IKK activity was expressed as the % of the control without EGCG3″Me. (D) NF‐κB‐mediated transcriptional activity was measured with or without EGCG3″Me (30 μm). Plasmid pNFkB‐TA‐Luc (0.4 μg) and the pGL4.74[hLuc/TK] plasmid (40 ng) were transfected into mouse POBs, and the POBs were cultured for 24 h with or without LPS (1 ng·mL^−1^) and EGCG3″Me (30 μm). The luciferase activity was measured with the Dual‐luciferase Reporter Assay system. A significant difference between the two groups was indicated; **P < 0.01 and ***P < 0.001 vs. control, ^##^ *P <* 0.01 and ^###^ *P *< 0.001 vs. LPS.

We previously reported that PGE_2_ production by osteoblasts is essential for inflammatory bone resorption induced by LPS 7. As EGCG3″Me suppressed LPS‐induced bone resorption, as shown in Fig. 1, we next examined the effects of EGCG3″Me on PGE_2_ production and the expression of the related enzymes in mouse osteoblasts using real‐time PCR. LPS (1 ng·mL^−1^) induced PGE_2_ production and the mRNA expression of COX‐2 and mPGES‐1, key enzymes for PGE_2_ synthesis, and adding EGCG3″Me (30 μm) clearly suppressed LPS‐induced PGE_2_ production and expression of COX‐2 and mPGES‐1 (Fig. 2A,B). LPS is also known to induce the expression of RANKL mRNA in osteoblasts, and EGCG3″Me clearly inhibited the LPS‐induced expression of RANKL mRNA (Fig. 2B). To explore the molecular mechanism of EGCG3″Me action, we performed an IKK activity assay *in vitro* and found that EGCG3″Me directly suppressed the IKK enzyme activity (Fig. 2C). Using a reporter gene assay for NF‐κB transcriptional activity, we found that EGCG3″Me significantly attenuated the LPS‐induced NF‐κB transcriptional activity (Fig. 2D). These data suggest that EGCG3″Me suppresses the LPS‐induced COX‐2/mPGES‐1‐mediated PGE_2_ production via the negative regulation of NF‐κB signaling in osteoblasts, resulting in the inhibition of osteoclast differentiation and bone resorption.

### Effects of EGCG3″Me on osteoclastogenesis from macrophages

**Figure 3:**
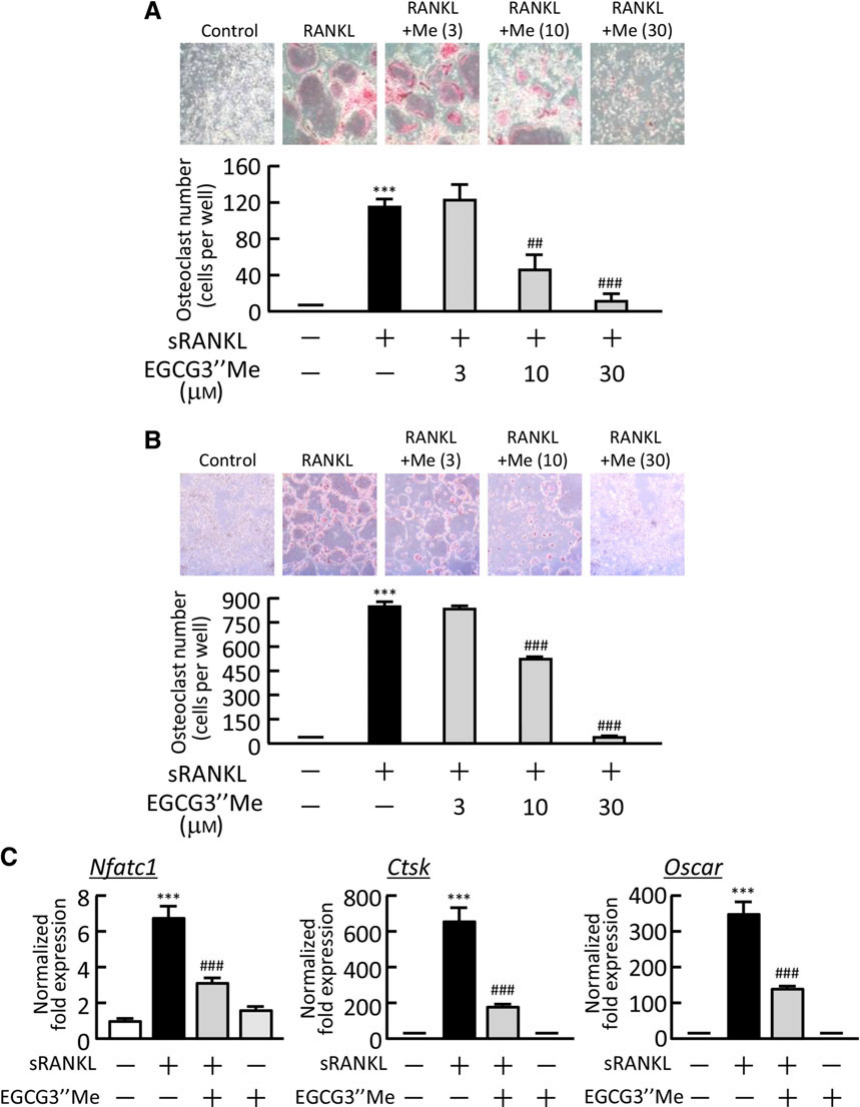
The effects of EGCG3″Me on osteoclast differentiation in osteoclast precursor cells. (A) Mouse bone marrow cells were cultured for 3 days with M‐CSF (100 ng·mL^−1^), and the BMMs formed were cultured for a further 5 days with M‐CSF (100 ng·mL^−1^) and sRANKL (100 ng·mL^−1^) in the presence or absence of EGCG3″Me (3–30 μm). The upper panel shows the TRAP‐stained cells in each cultured well. TRAP‐positive multinuclear osteoclasts were counted. The data are expressed as the means ± SEM of four wells. (B) RAW264.7 cells were cultured for 5 days in the presence or absence of sRANKL (100 ng·mL^−1^) and EGCG3″Me (3–30 μm). The upper panel shows TRAP‐stained cells in each cultured well. TRAP‐positive multinuclear osteoclasts were numbered. The data are expressed as the means ± SEM of three wells. (C) The mRNA expression of NFATc1 (*Nfatc1*), cathepsin K (*Ctsk*), and OSCAR (*Oscar*) in cultured RAW264.7 cells was analyzed by real‐time PCR. The data are expressed as the means ± SEM of three replicated wells in triplicate. A significant difference between the two groups was indicated; ***P < 0.001 vs. control, ^##^ *P* < 0.01 and ^###^ *P *< 0.001 vs. LPS.

To assess the direct action of EGCG3″Me on osteoclast precursor cells, BMMs were generated from bone marrow cells by culturing with M‐CSF (100 ng·mL^−1^) and then further cultured for 5 days with sRANKL (100 ng·mL^−1^) in the presence or absence of EGCG3″Me. EGCG3″Me (10 and 30 μm) dose dependently inhibited the RANKL‐induced osteoclastogenesis (Fig. 3A). In the cultures of the osteoclast precursor cells RAW264.7, adding EGCG3″Me (10 and 30 μm) suppressed RANKL‐dependent osteoclast differentiation (Fig. 3B). The mRNA expression of osteoclast‐specific genes, such as *Nfatc1*, cathepsin K (*Ctsk*), and *Oscar*, was greatly elevated in association with RANKL‐induced osteoclast generation in cultures of RAW264.7 cells. Adding EGCG3″Me significantly suppressed the expression of all of these genes in a real‐time PCR analysis (Fig. 3C).

### Effects of EGCG3″Me on alveolar bone loss in a mouse model of periodontitis

**Figure 4:**
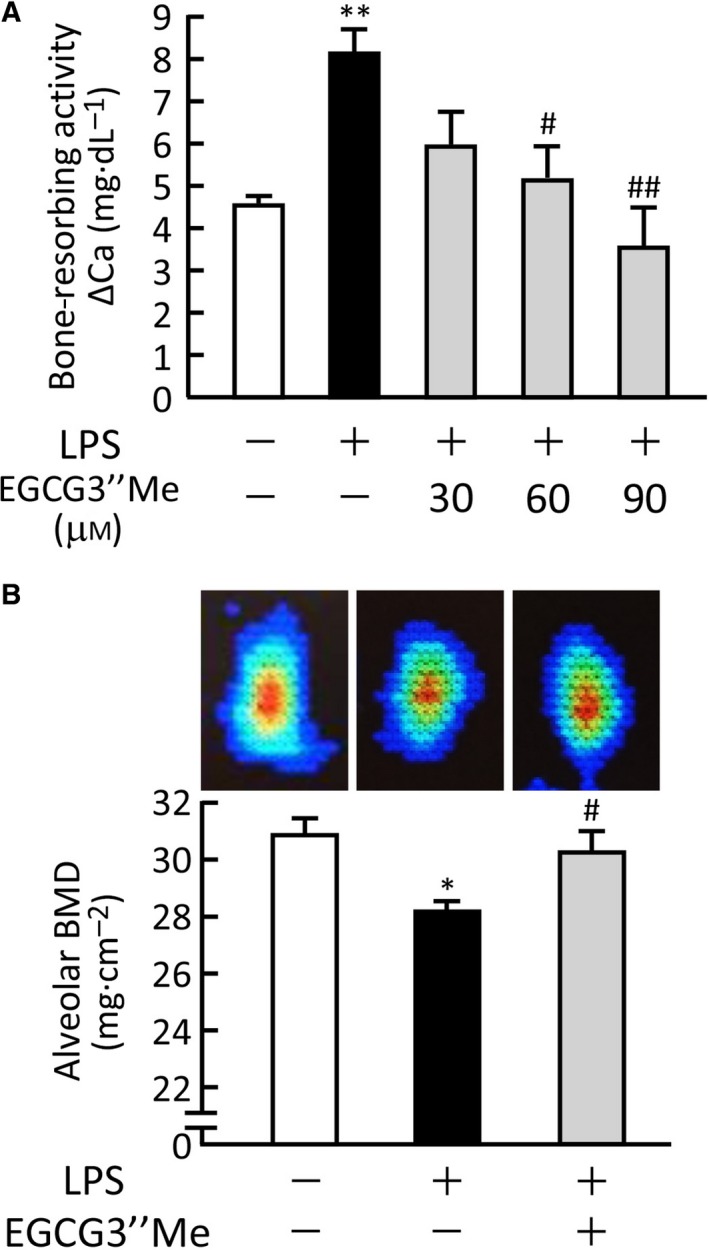
*O*‐methylated (−)‐epigallocatechin gallate attenuated the LPS‐induced loss of mandibular alveolar bone in mice. (A) Alveolar bone collected from mouse lower mandible was cultured for 5 days with LPS (5 μg·mL^−1^) and EGCG3″Me (30–90 μm). The bone‐resorbing activity was measured based on the calcium in the medium. The data are expressed as the means ± SEM of four cultures. (B) As a model for experimental periodontitis, LPS (25 μg per mouse) was injected with or without EGCG3″Me (100 μg per mouse) into the lower gingiva in mice on days 0, 2, and 4. The mandibular alveolar bones were collected on day 7 and measured for the BMD. The data are expressed as the means ± SEM of six or seven mice. Upper panels indicate the image data of DXA in each experimental condition. A significant difference between the two groups was indicated; *P < 0.05 and **P < 0.01 vs. control, ^#^ *P *< 0.05 and ^##^ *P* < 0.01 vs. LPS.

To determine whether or not EGCG3″Me exerted bone protective effects against periodontitis, we established alveolar bone organ cultures as an *in vitro* experimental model of periodontitis. Alveolar bones were collected from the mouse mandible and cultured for five days with or without LPS and EGCG3″Me. LPS‐induced alveolar bone resorption was significantly suppressed by adding EGCG3″Me (Fig. 4A). We further examined the effects of EGCG3″Me on alveolar bone loss using an *in vivo* mouse model of periodontitis. The injection of LPS in the lower gingiva induced the loss of BMD of alveolar bone, but co‐injection of EGCG3″Me with LPS restored the LPS‐induced alveolar bone loss (Fig. 4B). The image data of DXA clearly showed the loss of alveolar bone by LPS injection, and EGCG3″Me recovered the bone loss to the control level, as shown in the upper panels of Fig. 4B.

## Discussion

(−)‐Epigallocatechin‐3‐*O*‐gallate has been reported to exert various biological functions, including antioxidant, antiallergy, anticancer, and antibacterial activities 10, 11, 17. EGCG3″Me is known to be contained in some green tea cultivates, such as the Japanese tea Benifuuki. The biological functions of EGCG3″Me are similar to those of EGCG, but the potency of these EGCGs in different disease models is controversial.

In this study, we showed that EGCG3″Me inhibited LPS‐induced osteoclast differentiation and bone resorption in organ cultures of mouse calvariae (Fig. 1). In our previous study, the treatment of 30–90 μm EGCG dose dependently suppressed LPS‐induced bone resorption 15, but 3–10 μm EGCG3″Me clearly suppressed the bone resorption in the present study. Therefore, the inhibitory potency of EGCG3″Me seems to be slightly stronger than that of EGCG in bone resorption. In a mouse model of periodontitis, LPS‐induced bone resorption was suppressed by EGCG3″Me in organ culture of alveolar bone, and the alveolar bone loss was attenuated by the injection of EGCG3″Me into the lower gingiva *in vivo*. The potency of EGCG3″Me in this model is slightly stronger than that of EGCG 15. The mode of retention of EGCG3″Me in the gingival tissues must be examined in order to compare the effects of EGCG3″Me and EGCG in this model.

A previous study reported that the 67‐kDa laminin receptor (67LR) is a receptor for catechins, EGCG, and EGCG3″Me, but other catechins, such as catechin, epicatechin, and epigallocatechin, cannot bind to the 67LR 18, 19. The binding activity of EGCG to the 67LR was reported to be higher than that of EGCG3″Me, but the administration of EGCG3″Me resulted in stronger effects than that of EGCG in animal studies 20, 21. Further studies are needed to define the role of 67LR in the action of EGCG3″Me in target tissues.

In one clinical trial, the intake of Benifuuki tea showed stronger antihypertensive effects than Yabukita tea, which does not contain EGCG3″Me 20. In a mouse type IV allergy model, the antiallergy activity of EGCG3″Me by oral administration was higher than that of EGCG 13. Furthermore, Benifuuki tea extract exhibited a stronger antiobesity effect than did Yabukita tea extract in a mouse model of high‐fat diet obesity 22. These results suggest that the 3‐*O*‐methylation enhances EGCG's biological functions *in vivo*. However, the binding activity of EGCG3″Me to 67LR, a receptor for catechins, was lower than that of EGCG 19. Oritani *et al*. 21 examined the plasma concentration profiles of EGCG3″Me and EGCG after oral administration and found that EGCG3″Me maintained a higher plasma concentration than EGCG, and the bioavailability of EGCG3″Me was 2.7‐fold higher than that of EGCG in rats. Therefore, the potent activity of EGCG3″Me *in vivo* is probably based on its higher plasma concentration due to its efficient absorption from the intestine.

We previously reported that LPS recognizes TLR4, an LPS receptor in osteoblasts, and that LPS‐TLR4 signaling induces PGE_2_‐mediated bone resorption 7. Kumazoe *et al*. 23 reported that EGCG suppressed the expression of TLR4 in adipose tissues and obesity‐induced inflammation, suggesting that TLR4 may be a target molecule for EGCG and EGCG3″Me. In LPS‐TLR4 signaling, NF‐κB is a critical molecule for the subsequent transcription. NF‐κB binds to an inhibitor of NF‐κB (IκB)α and localizes to the cytosol without stimulation. On exposure to inflammatory stimuli, LPS‐TLR4 signaling activates the IKKα/IKKβ/NEMO (IKKs) complex, and IKKs lead to the dissociation of IκBα/NF‐κB complex. NF‐κB then shifts to the nucleus to activate the transcription of various target genes. In the present study, EGCG3″Me suppressed the mRNA expression of COX‐2, mPGES‐1, and RANKL in osteoblasts, and the transcription of these genes is known to be regulated by NF‐κB. Indeed, EGCG3″Me directly suppressed both the enzyme activity of IKK and the NF‐κB‐mediated transcriptional activity (Fig. 2C,D). Therefore, NF‐κB may be a critical molecule to consider when discussing the mechanism underlying the activity of EGCG3″Me in bone tissues.

As a model of periodontitis, we used a mouse model and showed that LPS injection into the lower gingiva induces the loss of alveolar BMD. Ossola *et al*. 24, 25 have shown the LPS‐induced periodontitis in rats and reported the beneficial effects of treatment with methanandamide and cannabinoid‐2 receptor agonist, which may be useful for the oral health. Gurkan *et al*. 26 also used the rat experimental periodontitis induced by LPS and reported the therapeutic effects of vasoactive intestinal peptide by monitoring alveolar bone. It is known that the pathological mechanism of human periodontitis is complex; however, the LPS‐induced experimental models using rats and mice are useful to monitor various beneficial compounds for periodontitis.

In cancer cells, previous studies have shown that EGCG binds to Fas and induces Fas‐mediated apoptosis 27 and that it inhibits the phosphorylation of vimentin, an intermediate filament, to suppress cell proliferation 28. Nomura *et al*. 29, 30 examined the mechanism underlying the activity of EGCG in carcinogenesis and reported that EGCG suppresses Akt activity in the UVB‐induced PI3K pathway and also inhibits TPA‐induced NF‐κB activation in mouse epidermal cells. Therefore, the target molecules for the action of EGCG and EGCG3″Me are different in each cell, but kinases such as IKK and Akt may be potential molecules for these EGCGs in target cells.

Using a mouse experimental model of periodontitis, we previously showed the potential effects of the green tea catechin EGCG in preventing alveolar bone loss 15. Here, we showed that EGCG3″Me also prevents alveolar bone loss in mice. Cho *et al*. 31 reported the effects of orally administered EGCG on ligature‐induced periodontitis in rats. As the bioavailability of EGCG3″Me is higher than that of EGCG, the oral administration of the green tea Benifuuki containing EGCG3″Me may be effective for preventing periodontitis, but further studies, such as clinical trials, are needed in order to define the role of EGCG3″Me in the pathogenesis of human periodontitis.

## Author contributions

TT, CM, and MI conceived and designed the experiments. TT, RI, SY, and KW performed the experiments. TT, CM, MH, and FMWG analyzed the data. CM, MI, and MH contributed reagents/materials/analysis tools. TT wrote the manuscript. CM, MI, and FMWG reviewed and improved the manuscript.
